# A detailed observation of the ejection and retraction of defense tissue acontia in sea anemone (*Exaiptasia pallida*)

**DOI:** 10.7717/peerj.2996

**Published:** 2017-02-21

**Authors:** Julie Lam, Ya-Wen Cheng, Wan-Nan U. Chen, Hsing-Hui Li, Chii-Shiarng Chen, Shao-En Peng

**Affiliations:** 1 Department of Biology, University of Ottawa, Ottawa, Ontario, Canada; 2 Graduate Institute of Marine Biology, National Dong Hwa University, Pingtung, Taiwan; 3 Department of Biological Science and Technology, I-Shou University, Kaohsiung, Taiwan; 4 Taiwan Coral Research Center, National Museum of Marine Biology and Aquarium, Pingtung, Taiwan; 5 Department of Marine Biotechnology and Resources, National Sun Yat-Sen University, Kaohsiung, Taiwan

**Keywords:** Acontia, *Exaiptasia pallida*, Defense, Ejection, Retraction

## Abstract

Acontia, located in the gastrovascular cavity of anemone, are thread-like tissue containing numerous stinging cells which serve as a unique defense tissue against predators of the immobile acontiarian sea anemone. Although its morphology and biological functions, such as defense and digestion, have been studied, the defense behavior and the specific events of acontia ejection and retraction are unclear. The aim of this study is to observe and record the detailed process of acontia control in anemones. Observations reveal that the anemone, *Exaiptasia pallida*, possibly controls a network of body muscles and manipulates water pressure in the gastrovascular cavity to eject and retract acontia. Instead of resynthesizing acontia after each ejection, the retraction and reuse of acontia enables the anemone to respond quickly at any given time, thus increasing its overall survivability. Since the *Exaiptasia* anemone is an emerging model for coral biology, this study provides a foundation to further investigate the biophysics, neuroscience, and defense biology of this marine model organism.

## Introduction

Like many cnidarians, sea anemones contain specialized cells, known as cnidocytes or nematocytes, in their body column, oral disk, pharynx, tentacles, mesenterial filaments, and acontia (Manuel, 1988; Shick, 1991). Cnidocytes contain nematocysts, penetrative cells that discharge toxic compounds into the target (Lotan et al., 1995) in response to certain chemical and mechanical stimulation (Mariottini & Pane, 2010). Despite being a venomous organism, *Exaiptasia* anemone are prey to *Lysmata* shrimp and nudibranch species, such as *Aeolidiella stephanieae* (Valdés, 2005). In order to protect themselves from predator attacks, anemones either detach and move by pedal locomotion or eject acontia by contracting the body column and extending its tentacles in self-defense (Waters, 1973; Edmunds et al., 1974; Edmunds et al., 1976; Schlesinger et al., 2009). Based on our observation, *Exaiptasia* anemones rarely perform detachment and pedal locomotion, but rather the ejection of acontia.

**Figure 1:**
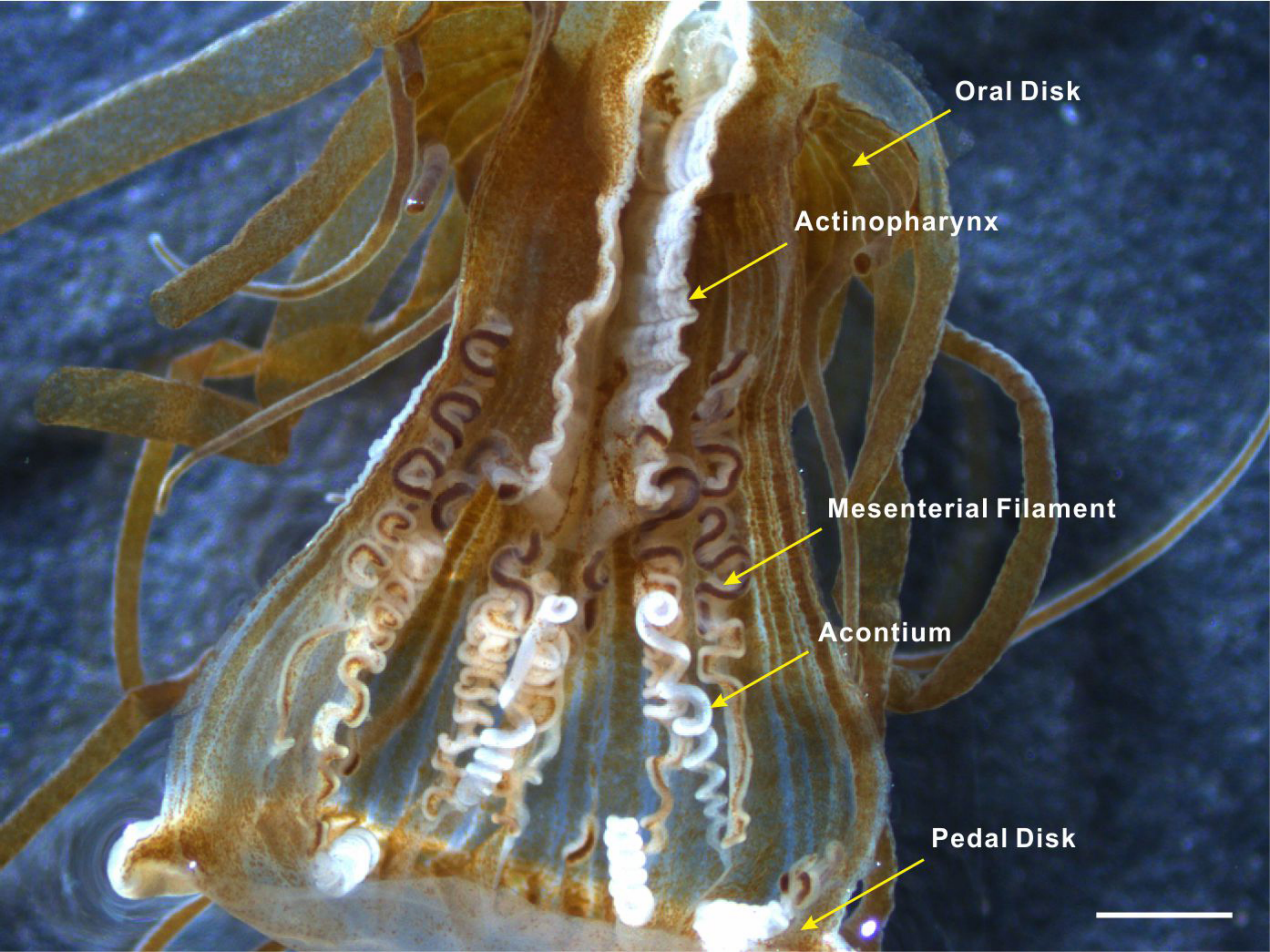
Acontia tissue within the body column of *Exaiptasia* anemone. Acontia tissue is comprised of long white thread-like organs that have a simple coiling morphology; this tissue is densely lined with nematocysts and form from the mesentery edge near the pedal disk of the anemone. Scale bar: 1 mm.

Acontia is the distinguishing feature of the actiniarian group Acontiaria (Rodríguez et al., 2012). Acontia in sea anemones usually have a white, coiled threadlike appearance and form at the end of the thickened edge of mesenteries near the pedal disk (Fig. 1). These thread-like extensions of the mesenterial filaments are filled with nematocyst-containing cnidocytes (Stephenson, 1935). Nematocyst discharge can be induced by physical contact, specific molecules, or chemical markers that are possibly recognized by a cellular recognition system of the anemone (Yanagita, 1959; Yanagita, 1960; Blanquet & Lenhoff, 1966; Ishihara, 1967; Blanquet, 1970; Lubbock, 1980). Once stimulated, the dart-like tubules are propelled from the nematocysts with enough force to penetrate the exoskeleton of the predator to sting its target with cytolytic peptide and protein toxins that cause paralysis (Conklin, Bigger & Mariscal, 1977; Kem, 1988; Bernheimer, 1990; Turk, 1991; Maček, 1992; Anderluh & Maček, 2002). Acontial defenses of the anemone can sometimes dissuade *Aeolidia papillosa* from feeding and potentially result in the death of this nudibranch (Harris, 1973). Since detachment and pedal locomotion have rarely been observed in *Exaiptasia* anemones, acontia ejection serves as an important defense mechanism against predator attack.

Early reports have briefly described the phenomenon of acontia ejection, whereby the threatened anemone strongly contracts its body column, forcing water out of the cinclides. This causes the ejection of acontia, which are carried by the water currents (Stephenson, 1928; Manuel, 1988). However, the details of acontia control, specifically retraction, are unclear. With merely the presence of nervous elements but no nerve net or brain (Wada, 1972), the anemone is able to control the ejection and retraction of acontia using the shortening or elongation of the body column in response to mechanical stimulation. These simple but effective control mechanisms of acontia enable the anemone to readily respond to predation at any given time. Through detailed observation, this study presents meticulous detail of acontia ejection and retraction during the defensive and recovering states of anemones.

## Material and Methods

The sea anemones, *Exaiptasia pallida,* were collected from the tanks in the Husbandry Centre of the National Museum of Marine Biology and Aquarium in Pingtung, located in Southern Taiwan. The origin of the anemones comes from the wild population, since the unfiltered seawater was pumped from the location beneath the native habitat (N22 03 00.08 E120 41 42.88) of *Exaiptasia pallida* (the scientific name was recently changed from *Aiptasia pulchella* by Grajales & Rodríguez 2014). Collected anemones were cultured in tanks with filtered seawater at an ambient temperature (25 °C) with a 12 h light (34 µmol m^−2^s^−1^): 12 h dark photoperiod in laboratory. Anemones were fed *Artemia* nauplii weekly. Samples with a body column height greater than 20 mm were chosen for this study due to the positive correlation of the effectiveness of acontial ejection with the size of the anemone (Harris, 1986). Samples were removed by scraping beneath the pedal disk to detach the anemone from the tank. The anemones were cultured in tanks separately for one week before observation. During observation, a plastic dropper was used as a seemingly threatening stimulus to provoke the anemone to exhibit defensive behavior. For each stimulation, we probed the anemone between the cinclides and oral disk several times for about two to three seconds until the contraction of the body column into a ball-like shape. Then, we waited for the anemone to relax by extending its tentacles, before continuing with the next stimulation. After allowing the anemone to recover slightly, we proceeded with the second stimulation to the target region, inducing the ejection of acontia. Repeated stimulations were performed to induce more acontia ejection from the cinclides. The ejection and retraction process of six specimen were observed and recorded. High quality videos (Video S1) were recorded using a high definition camcorder (HDR CX-550; Sony, Tokyo, Japan). Snapshot images were taken from the recorded video materials, and measurements were made using the open source, Java-based software package Image J (National Institutes of Health, Bethesda, MD, USA).

**Figure 2:**
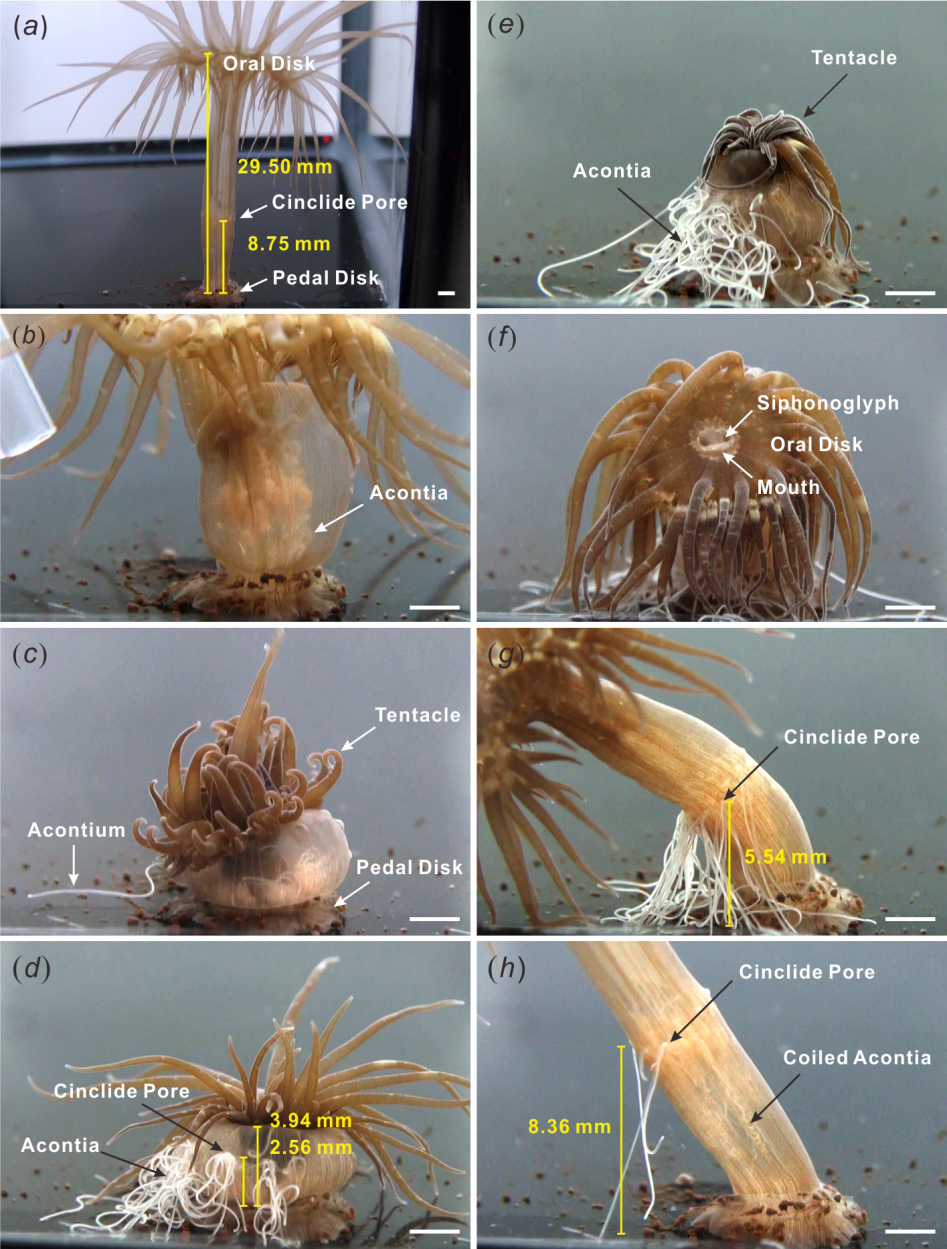
Acontia ejection and retraction. (A) relaxed anemone, fully elongated; (B) initial stimulation made the anemone contract but did not release of acontia; (C) the second stimulation caused slight acontia ejection; (D) multiple stimulations provoked defensive state with the profuse release of acontia and upright tentacles; (E) anemone displayed deflated morphology and withdrawn oral disk; (F) start of recovery, anemone mouth remained closed while siphonoglyphs were opened for the intake of water; (G) column elongation and acontia retraction; (H) approximately 40–50 min for the retraction of all acontia until next ejection (Video S1). Scale bar: 2 mm.

## Results

In this study, the relaxed adult anemone (*Exaiptasia pallida*) displayed an elongated body column with tentacles evenly spread out at the oral disk. In Fig. 2A, the length of the body column from the oral disk to the pedal disk of the representative anemone was 29.50 mm (average =30.65 ± 2.90 mm (SEM for this and all other error terms), *n* = 6 specimens, Table 1), and the approximate distance between the cinclides and the pedal disk was 8.75 mm (average =7.73 ± 0.73 mm (SEM), *n* = 6 specimens, Table 1). When the anemone was gently probed with a plastic dropper, no acontia were released. The anemone only withdrew by shortening its body length (Fig. 2B).

**Table 1:** The height of cinclides during acontia ejection and retraction.

Specimen no.	Height of body column (mm)			Height of cinclide (mm)			Ratio	
	Initial^a^	Ejection^b^	Ratio^(b/a)^	Initial^c^	Ejection^d^	Retraction^e^	Ejection^(d/c)^	Retraction^(e/d)^
1	29.50	3.94	0.134	8.75	2.56	8.36	0.293	3.266
2	23.87	7.39	0.310	6.46	4.95	7.91	0.766	1.598
3	29.83	3.08	0.103	6.44	2.48	3.51	0.385	1.415
4	25.40	4.18	0.165	7.13	1.59	6.07	0.223	3.818
5	31.42	4.59	0.146	6.63	2.70	8.30	0.407	3.074
6	43.90	5.34	0.122	10.94	2.23	3.70	0.204	1.659
Average ± SEM	30.65 ± 2.90	4.75 ± 0.61	0.163 ± 0.031	7.73 ± 0.73	2.75 ± 0.47	6.31 ± 0.92	0.380 ± 0.084	2.472 ± 0.422

The second stimulation, during which the body column of the anemone was repeatedly probed with a dropper, resulted in the ejection of some acontia (Fig. 2C). Repeated stimulation to the target area caused the anemone to further contract into a ball-like shape with fully extended tentacles (Fig. 2D, Video S1). The length of the body column became 3.94 mm (average =4.75  ± 0.61 mm (SEM), *n* = 6 specimens, Table 1), and the distance between the cinclides and the pedal disk was approximately 2.56 mm (average =2.75 ± 0.47 mm (SEM), *n* = 6 specimens, Table 1). As the anemone contracted its body column, acontia were profusely released from cinclides (Fig. 2D, Video S1) and simultaneously resulted in the deflation of the tentacles (Fig. 2E). At this stage, the height between the cinclides and the base of a representative anemone decreased by 29.3% (average = 38 ± 8.4% (SEM), *n* = 6 specimens, Table 1).

After the removal of all physical stimuli, the anemone slowly recovered. The body column was restored to a relaxed state of an exposed oral disk and extended body column and tentacles (Fig. 2F). In its relaxed state, the mouth was closed and siphonoglyphs widely opened to limit and control water intake. The acontia were retracted into the anemone as the body column slowly extended to a length of 5.54 mm from the cinclides to the base (Fig. 2G). Eventually, the distance between the cinclides and the base of the representative anemone increased to 8.36 mm (average =6.31 ± 0.92 mm (SEM), *n* = 6 specimens, Table 1) due to the body column becoming fully elongated; at this point, the acontia recoiled inside the body (Fig. 2H). During the recovery process, the height between the cinclides and the base of the representative anemone increased by 327% (average = 247 ± 42.2% (SEM), *n* = 6 specimens, Table 1). The entire acontia recovery process took about 40–50 min for the representative anemone (Video S1).

## Discussion

*Exaiptasia* anemones are simple bi-radial organisms that are exclusively polypoid with an external morphology limited to tentacles, oral disk, mouth, body column, and pedal disk (Manuel, 1988; Shick, 1991) without a central nervous system (Dahl et al., 1963; Nakanishi et al., 2012). External stimuli are sensed by a nerve net that extends throughout the body. The nerve net follows the network of contractile muscle systems, which contain sensory neurite cells between the endoderm and muscle fibers (Batham, Pantin & Robson, 1960). Passing through the mesoglea, neurites connect to muscles in the ectoderm and endoderm for coordination and rapid response (Robson, 1963).

When initially stimulated by a dropper, the *Exaiptasia* anemone contracted its body column (Video S1). This is a common response of anemones to external stimuli, such as water flow changes and physical contact. The degree of body contortion depends on the amount of contraction exerted by longitudinal muscles in the body column and retractor muscles in the mesenteries. Since the hydrostatic skeleton determines the body shape of the anemone (McFarlane, 1974), any change in water pressure will alter the shape of the anemone. Thus, we hypothesize that the contraction of the body column of the *Exaiptasia* anemone generates positive water pressure as demonstrated in the *Metridium* anemone (Batham & Pantin, 1950), forcing water out through the cinclides, which carries the free ends of the acontia with it (Manuel, 1988), and causes the deflation of the tentacles.

Mimicking the possible attack of a predator, repeated mechanical stimulation provoked the anemone to exhibit defensive behavior, which resulted in an average decrease of body length by 0.163 ± 0.031 fold (Table 1). The defensive behavior is characterized by the compression of the body, extension of tentacles, and ejection of acontia. This form enables the anemone to shield the oral disk using cnidocytes in the tentacles and protect the body using acontial stinging cells. This behavior may deter the predator, while the ejection of acontia act as a first line of defense by stinging the predator before close contact (Edmunds et al., 1974; Edmunds et al., 1976). Since in its natural environment, *Exaiptasia* anemones are attacked by *Aeolidia papillosa* and *Lysmata wurdemanni*, which feed by repeatedly poking, prodding, or biting the body column close to the pedal disk of the anemone (Edmunds et al., 1974; Edmunds et al., 1976; Rhyne & Lin, 2006), the control mechanism of acontia is a crucial factor for its survival.

Based on this study, we propose that the control of rapid acontia ejection potentially involves two factors: the contraction of the body column and the generation of positive water pressure in the gastrovascular cavity. The relaxation of circular muscles allows a wider body, while the contraction of longitudinal muscles contorts the body column. Next, the contraction of the retractor muscles withdraws the oral disk. The compressed oral disk halts water intake causing the anemone to become a closed system (Josephson, 1966). The contraction of muscles forces the body to contort vertically and effectively propel water out and eject acontia via cinclides for defense (Fig. 2D, Video S1). Since muscle fibers within the acontia do not have directed movement or sensory mechanisms (Wada, 1972), it cannot eject effectively without this mechanism.

**Figure 3:**
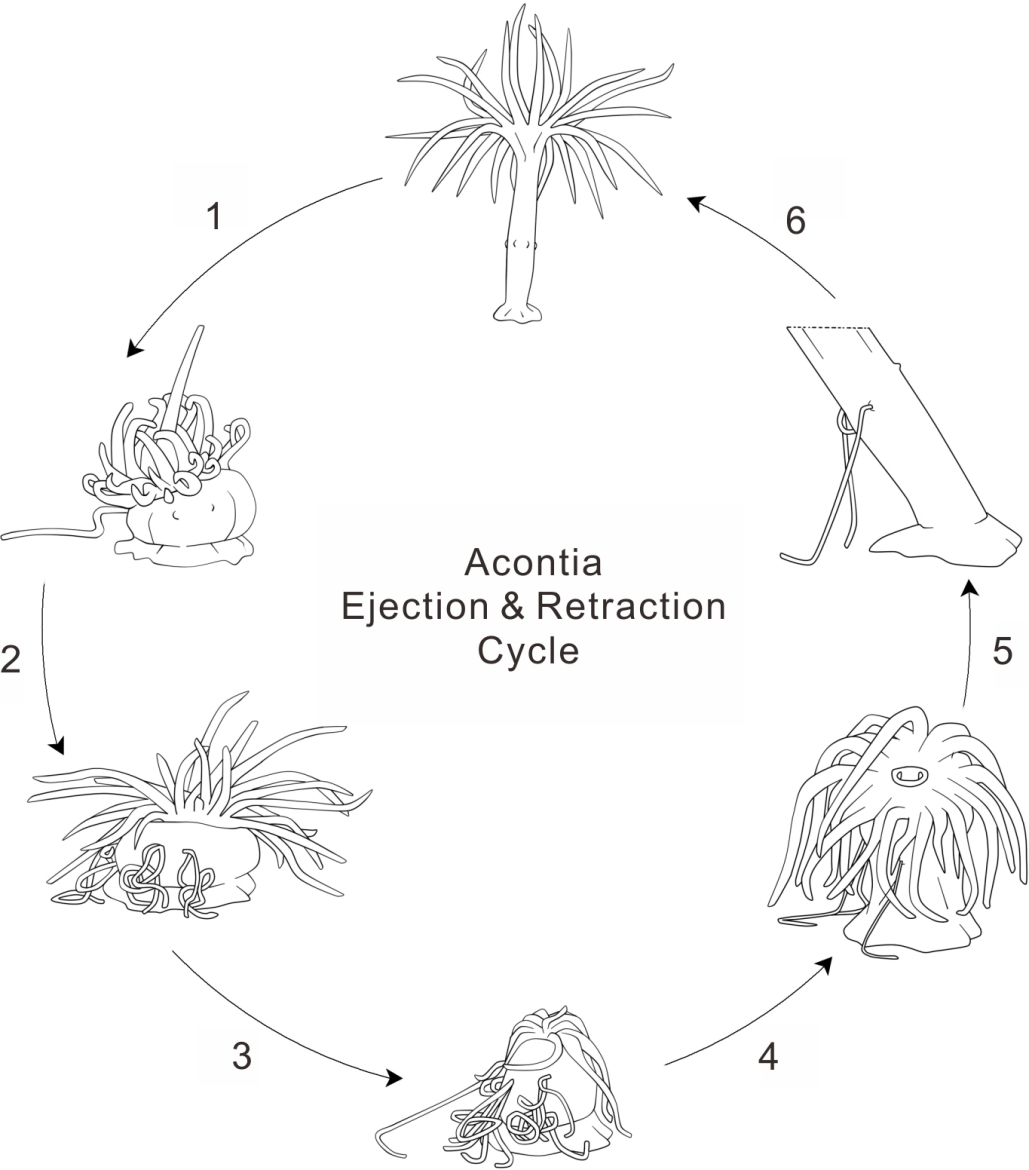
Anemone acontia ejection and retraction cycle. (1) first stimulation caused body contraction and increased water pressure; (2) repeated stimulations prompted anemone defensive state and acontia ejection; (3) deflated oral disk and tentacles for maximum water expulsion; (4) anemone entered recovery state when stimulations ceased; (5) open siphonoglyphs intake water and closed mouth hold water volume, tentacles inflated and oral disk exposed; (6) generation of negative water pressure and elongation of body column pulled acontia back into body, process lasted for 40–50 min.

During the recovery period, the anemone initially compresses the tentacles into a shriveled shape for maximal expulsion of water from the anemone body. Later, the anemone exposes the oral disk and extends the tentacles. Although the mouth is closed, the siphonoglyphs remain open for slow water intake (Video S1). With the relaxation of the longitudinal muscles and contraction of the circular muscles, water fills the gastrovascular cavity and extends the body column vertically (Batham & Pantin, 1950). This suggests that the control of acontia retraction involves two critical factors: the elongation of the body column and the generation of negative water pressure. Negative water pressure was observed in the *Metridium* anemone when recovering from a state of extreme contraction (Batham & Pantin, 1950). This negative water pressure in the gastrovascular cavity results in the intake of water through the siphonoglyphs and cinclides (Figs. 2E and 2F). The slow suction of water elongates the body column by filling up the total water volume. Although acontia can extend for many centimeters beyond the column wall (Manuel, 1988), acontia have a limited length. Thus, the elongation of the body column helps the retraction of acontia into the body, especially when the acontia is adhered to an immotile surface. This elongation is evident by the distance measured from the cinclides to the base of the tank, which increased by an average of 2.472 ± 0.422 fold (Table 1). As acontia are retracted, they recoil due to the unique position of acontial longitudinal muscles (Wada, 1972). In its natural environment, however, some of the anemone’s acontia may not be retracted when they are ripped off by the predator.

Acontia make up a unique defense tissue in acontiarian sea anemones. The ejection of acontia is an interesting process; acontia protrude through the body wall of cnidarian species upon the attack of predators or artificial stimulation. This study increases knowledge of the anemone’s defense behavior. For the first time, this study shows that the sea anemone possibly controls a network of body muscles and manipulates water pressure in the gastrovascular cavity to eject and retract acontia as a defense response. This study also provides insight to the ingenious control and economical reuse of acontia (Fig. 3). Furthermore, as an emerging model animal for coral biology, the observations and supplementary video of this study provide important information that fuels the future study of biophysics, neuroscience and defense biology of the *Exaiptasia* anemone to unmask the amazing behavior of this marine model organism.

##  Supplemental Information

10.7717/peerj.2996/supp-1 Video S1 The ejection and retraction of defense apparatus acontia in sea anemone *Exaiptasia pallida*

## Additional Information and Declarations

### Data Availability

The following information was supplied regarding data availability: All of the raw data are presented in Table 1. A supplementary video has been included as Video S1.
